# Supplementary material for: Screening of Yeasts Isolated from Baijiu Environments for Producing 3-Methylthio-1-propanol and Optimizing Production Conditions
Source: Foods. 2022 Nov 12;11(22):3616. doi: 10.3390/foods11223616 (PMC9689521; doi:10.3390/foods11223616)
Supplement: Supplementary file 1 [file foods-11-03616-s001.zip › foods-1950396-supplementary.pdf]

Supplementary material for

Screening of yeasts isolated from *Baijiu* environments for producing 3-methylthiopropanol and optimizing production conditions

Supplementary Table S1. Factors and levels of single factor design and their optimization conditions.

Factor	Level/type	Optimization condition
Glucose concentration (g/L)	10, 20, 30, 40, 50, 60 and 70	**30-60**
Yeast extract concentration (g/L)	0, 0.4, 0.8, 1.2, 1.6, 2.0, 2.4 and 2.8	**2-2.8**
L-Met concentration (g/L)	0, 2, 4, 6, 8, 10, 12 and 14	**4**
Time point of L-Met addition (h)	0, 12, 24, 36, 48, 60 and 72	**24**
Initial pH	3.0, 3.5, 4.0, 4.5, 5.0, 5.5, 6.0, 6.5 and 7.0	**4.0-5.5**
Temperature (℃)	20, 24, 28, 32, 36 and 40	**32**
Shaking speed (rpm)	0, 45, 90, 135, 180, 225 and 270	**135**
Loading volume (mL/250 mL)	25, 50, 75, 100 and 125	**75**
Inoculum size (%, v/v)	0.1, 0.2, 0.4, 0.8, 1.6, 3.2 and 6.4	**0.2-3.2**
Surfactant types	Control, glycerol, Tween-20, Tween-40, Tween-60, Tween-80, Triton X-100 and Triton X-114	**Tween-20-80 and Triton X-100**
Tween-80 concentration (g/L)	0, 2, 4, 8, 16, 32 and 64	**2-64**
Time (h)	0, 12, 24, 36, 48, 60, 72, 84 and 96	**48-96**

Supplementary Table S2. Physiological and biochemical characteristics of strain YHM-G.

Tests	Results	
	**Sugars**	**Characteristics of YHM-G**
**Sugar fermentation tests**	Glucose<br>Maltose<br>D-Galactose<br>Sucrose	Acid and gas production
	D-Maltose	Acid production, no gas
	D-Xylose	Gas production, no acid
	L-rhamnose monohydrate<br>D-Arabinose<br>Lactose	No acid, no gas, but grow
	**Carbon sources**	**Characteristics of YHM-G**
**Carbon source assimilation tests**	Ethanol<br>Glycerol<br>Inulin	+
	D-Raffinose<br>D-Trehalose<br>Mannose<br>D-Ribose<br>D-Sorbose	−
	**Nitrogen sources**	**Characteristics of YHM-G**
**Nitrogen source assimilation tests**	Urea<br>Ammonium sulfate<br>Sodium nitrite<br>Potassium nitrate<br>L-Phenylalanine<br>L-Lysine	+
**Other tests**	Indole test<br>Methyl red test<br>Starch hydrolysis test<br>Citrate test<br>Urea test	+
	Voges-Proskauer test<br>Hydrogen sulfide test<br>Gelatin liquidized test	−

Note: "+", positive response; "−", negative response.

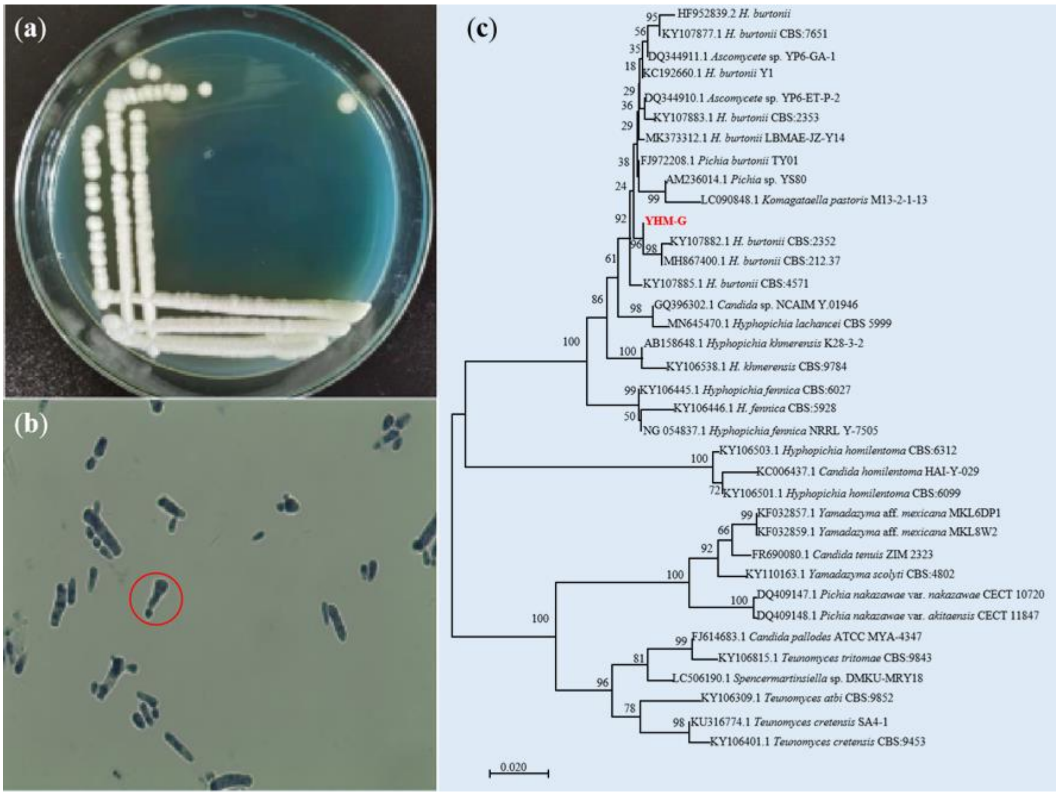

Supplementary Figure S1. Colony morphology on WL medium (**a**), cell morphology (10×100 times magnification) (**b**) and neighbor-joining phylogenetic tree based on ribosomal large subunit 26S rRNA gene (D1/D2 region) gene sequence and closest relative species (**c**) of strain YHM-G. Cell was stained by the methylene blue method. The asexual budding reproduction occurred at the ends of the cells was highlighted in the red circle.

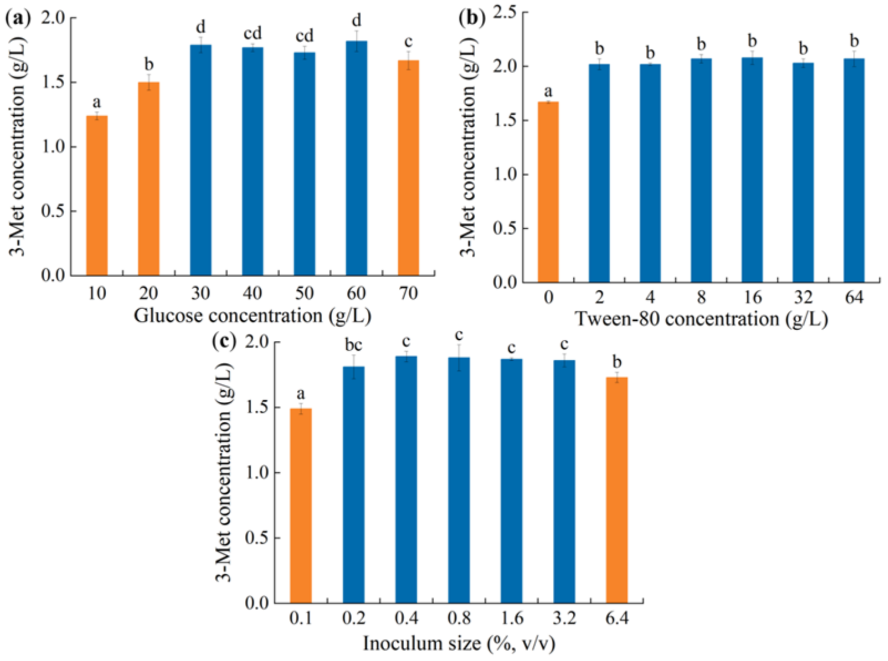

Supplementary Figure S2. Effect of glucose concentration (10, 20, 30, 40, 50, 60 and 70 g/L) (a), T-ween-80 concentration (0, 2, 4, 8, 16, 32 and 64 g/L) (b), inoculum size (0.1, 0.2, 0.4, 0.8, 1.6, 3.2 and 6.4%, v/v) on 3-Met concentration. Same letters in the column indicates that the data do not differ significantly at 5% probability by the Tukey test.

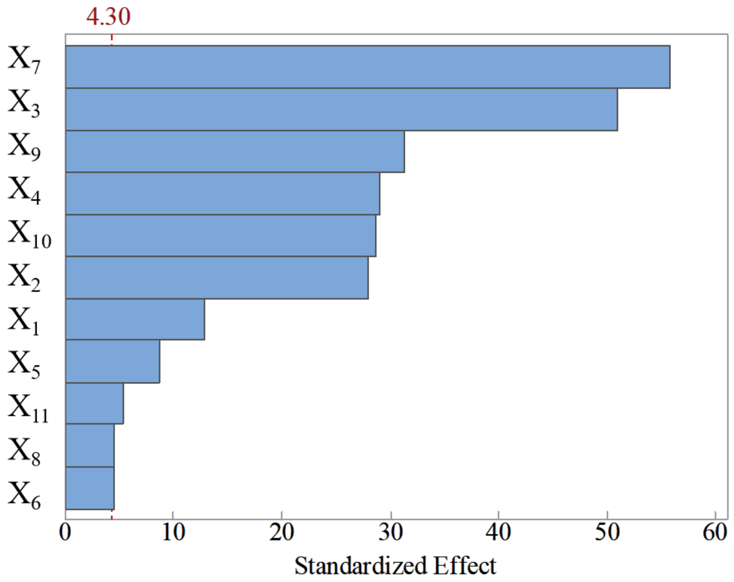

Supplementary Figure S3. Standardized Pareto chart showing the effect of variables on 3-Met concentration. X1, shaking speed; X2, temperature; X3, glucose concentration; X4, initial pH; X5, loading volume; X6, inoculum size; X7, time point of L-Met addition; X8, culture time; X9, yeast extract concentration; X10, L-Met concentration; X11, Tween-80 concentration.
